# Fostering Behavioural Change Towards Integrated Care – a Multi-Team Case Study in Specialised Youth Services

**DOI:** 10.5334/ijic.8842

**Published:** 2025-02-07

**Authors:** Andrée Sekreve, Maurits Struik, Woody van Olffen, Laura Nooteboom

**Affiliations:** 1 Youké Youth Care, 109 Verlengde Slotlaan, 3700AW Zeist, The Netherlands; 2 TIAS Business School | Tilburg University, The Netherlands; 3 AMI Consultancy, Rotterdam, PO Box 90153, 5000 LE, Tilburg, The Netherlands; 4 LUMC Curium-Department of Child and Adolescent Psychiatry, Leiden University Medical Center, Post Box 15, 2300 AA, Leiden, The Netherlands

**Keywords:** integrated care, change management process, child and adolescent mental health care, families

## Abstract

**Introduction:**

Integrated care is crucial in delivering coherent and coordinated support to families with multiple and complex problems. Reorienting care organisations towards integrated care is a complex organisational change process. It requires both structural and behavioural adjustments. To learn about effective practice, it is useful to study implementation between contexts.

**Description:**

This mixed methods case study provides a comparison over time of five regional teams simultaneously implementing an integrated care delivery mode. Group interviews identified whether and how different elements in the change approach helped or hindered the change progress.

**Discussion:**

We describe and discuss how the teams were guided and supported in learning to make the behavioural switches associated with their new integrated mode of operation.

**Conclusion:**

Our support-interventions appeared to be largely successful in fostering four pre-defined integrated care behaviours. This research took place during the Covid-19 pandemic, which was challenging but also brought unexpected benefits.

## Introduction

Families with complex and multiple problems across life domains are experiencing hardship in gaining access to appropriate care [1,2]. These families often experience fragmented care provided by various organisations [3,4]. Offering integrated care is often advocated as a fitting alternative to diminish the adverse consequences of fragmented care on families [5,6,7]. In this case study, integrated care is defined as ‘coherent, continuous, and coordinated support, organised across services, and wrapped around families’ needs [8,9].’ This case study describes the efforts of the Dutch youth care provider Youké to strengthen its specialised outpatient youth services by forming ‘integrated service teams’. In these teams, different youth care disciplines [e.g. family therapists, psychologists, social workers] with various expertise [e.g. trauma and attachment, psychopathology, complex divorce, systemic therapy] are combined as one team of professionals. Research shows [10] that forming such ‘multidisciplinary teams’ serves as a facilitator for effective integrated care because it stimulates interprofessional collaboration. This is essential for strengthening the resilience of professionals with different expertise to deliver integrated care in an often highly fragmented context.

However, to achieve a truly integrated approach for families, the structural intervention of just changing the composition of care providing teams is insufficient. For the structural change to be effective, behavioural changes in the daily practice of teams are needed as well. Specifically, effective implementation of integrated care depends on the active involvement and support of the workforce [11,12]. Few research projects to date have focused on how a complex structural change process can be effectively guided to achieve actual beneficial behavioural changes among professionals [2,12].

In this study we evaluate the interventions that were used to facilitate the switch to integrated care within five regional teams. Our aim is to identify key factors around those interventions that contributed to the behaviourally effective implementation of integrated care.

## Change context

### Study background

To make the shift towards integrated care at Youké, two implementation tasks had to be undertaken. The first task involved transforming and reshaping the regional teams of professionals within the organisation. This entailed changing teams consisting of professionals who worked solely within their own expertise or methodology, to collaborative, multidisciplinary teams of professionals with various expertise. This enables youth care professionals to consider the strengths and challenges of families from multiple professional perspectives to provide a broader, more holistic view on the context of families [12]. The second task was implementing an analysis model: the so-called ‘7-factors model’ [13]. This is a transdiagnostic case conceptualization method [14] designed to achieve a structured, coherent, clear analysis of the appropriate and desired care. In our case, the associated new behaviours to be learned by team members to achieve integrated care were fourfold: 1] consulting with colleagues with different expertise in a multi-disciplinary team setting; 2] performing a case conceptualisation to determine the most appropriate care for a family; 3] discussing this case conceptualisation in the multi-disciplinary team to consider the family’s case from different areas of expertise, and 4] leading colleagues towards working in multi-expertise teams.

#### Organisational background

Youké is a not-for-profit youth care provider in The Netherlands, offering specialised treatment for children between 0–23 years of age. According to ‘The Dutch Central Bureau of Statistics’ [CBS] in 2023 one in every 9.3 young people up to the age of 23 has received youth care in the Netherlands [15]. Youké has about 580 employees and its mission is to strengthen children and families to continue their lives without professional support and with renewed confidence. The core values are ‘first time right’, ‘personal’, and ‘close’ [16]. The provided care consists of outpatient care, inpatient care, foster care, and day treatment.

Professional youth care in the Netherlands is coordinated on a regional level. The Netherlands has 42 youth care regions across twelve provinces. Youké is active in eight regions, within the provinces of Utrecht, Gelderland and Noord-Holland. This study was carried out in five of the eight regions. The change was initiated by the management team of Youké and was led by a project team, consisting of the human resources manager, the quality manager, a quality policy advisor/researcher, and the project lead. The last two are also the first two authors of this paper.

### Implementation challenges

The implementation of integrated care is a complex process [10] demanding extensive and ongoing organisational support [17], in which involving and supporting the workforce should be a priority [11]. The change project within Youké started in 2020 with a focus group for professionals to introduce integrated care. Several obstacles to change in professional practices were identified. First, professionals found it difficult to grasp the implications of the change for their daily practice. Second, within the original teams, there were deeply ingrained working methods that professionals had trouble letting go of. This was caused by uncertainty about whether the change would actually be beneficial to daily practice. Teamleads described practical obstacles to implementing change, including limited time for professionals, concerns about meeting production standards and skepticism about the need for change, as some felt they were already providing integrated care. From this feedback, we concluded that reluctance to change among our colleagues could be attributed to a lack of understanding, urgency, perceived opportunity and professional engagement [18,19]. To them, it often felt like an abstract matter. For this planned change to succeed, we therefore deemed it necessary to create a more immediate and visible connection between the change project’s goals and the actual day-to-day behaviour of the professionals. In the end, sustained change in behaviours of professionals is the only criterion that shows lasting change is achieved [20].

**Figure 1:**
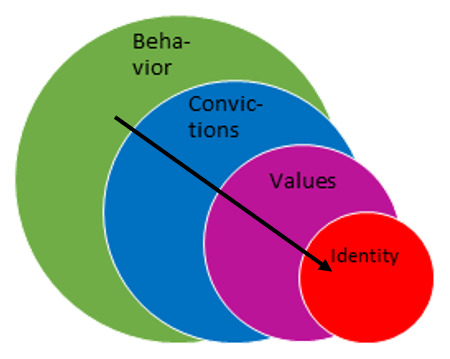
Change depth model [19].

Models of change depth [21,22 – see Figure 1] posit that for a large collective behavioural change to be durable, a concurrent matching shift in the ‘deeper’ underlying convictions, values and possibly even identities may be necessary. Behaviours, convictions, values, and identities should be re-aligned over time [23,24] for a new stable equilibrium to form. As such, a collective behavioural change of sufficient size usually requires a shift in underlying values – i.e. in culture – in order to be permanent and stable. Still, no matter how deep or cultural the change is, collective shifts in behaviour are a necessary condition for any real organisational change to occur. Therefore, the process of learning to adopt new behaviours and ending old, ingrained behaviours is central to any successful organisational change. In this study, we therefore examined to what extent professionals succeeded in switching to the desired behaviours of integrated care during the change process. Several supporting intervention steps were taken to stimulate learning of the desired changes. These interventions are described in the next paragraph.

### Change support intervention program

The support-intervention program was carried out between November 2020 and September 2021. The program was based on the ‘*Engage!* method [22], which largely builds on a dialogic organization development approach to transformation and change [25]. In dialogical approaches, the goal is to explore and encourage new thinking and ideas in the people who are agents in the change themselves instead of implementing a top down vison or plan. The various steps of the support-intervention program are displayed in Table 1.

**Table 1:** Change support-intervention program.


1. Forming change coalitions	Who: Teamleads, the project lead and policy advisor How: one meeting of 1 hour When: Q1 2021 Tools & working methods: Coalition formation What: Five change coalitions were made responsible for the design and implementation of the change in their own region. Each regional coalition was composed to consist of a mix of enablers, hotspot leaders and informal leaders [22]. *Enablers* were teamleads in the organisation with hierarchical power in the region. Each region nominated one teamlead for this task. This person was in turn responsible for putting together a change coalition with hotspot leaders and informal leaders. *Hotspot leaders* were professionals at crucial positions in the organisation with the ability to make the change a success, such as account managers or care coordinators. *Informal leaders* were professionals with a natural authority based on their expertise, personality or track record in the region. The size of each regional coalition depended on the number of full-time equivalents [FTEs] per region, ranging from five to eight professionals. The criteria for participation in the coalition was the positive motivation of the professional, and the teamlead’s assessment whether this professional would make an essential contribution to the change task.

2. Onboarding	Who: all members of the five change coalitions How: one meeting of 1,5 hour When: Q1 2021 Tools & working methods: coalition building, alignment creation, formal structuring/embedding, group dialogue. What: Each coalition received instruction by the project lead in one meeting of 1,5 hour about their role as change agent, and about the change approach: ‘the onboarding’. By discussing the project plan and progress of the change with the organisations’ management team, coalitions were also given a clear and strong mandate from the management team and the project lead for their role as change agents. At the start of the project the entire middle management was informed by two members of the project team in a meeting and had the opportunity to ask questions and raise concerns about the project and the change approach.

3. Learning expeditions	Who: all members of the five change coalitions How: 4 online meeting of 3 hours When: Q1 en Q2 2021 Tools & working methods: Visioning, *Golden Circle* [26], *Change Canvas* template [22], building a collective learning mindset [e.g. experimenting], broadening/extending the coalition base. What: The change coalitions were asked to design and define integrated youth care at Youké in a learning expedition. We applied methods in the spirit of dialogic organisation development [25] to facilitate collective learning. Due to Covid-19 measurements, four digital meetings took place digitally once a month, which lasted three hours each. In the first meeting we focused on the ‘why’ of the change to integrated care, to create a sense of need and common understanding among the change coalitions. In the second and third meeting, we paid attention to the ‘how’ and ‘what’ of the change process. During the fourth meeting, we discussed how to disseminate the change assignment from the level of the coalitions to the other professionals in the organisation and how they could be included in the change. The coalitions were then invited to organise expeditions within their own region, to start the dialogue about changing towards integrated care and to take the lead in implementing this further in their own region.

4. Coaching sessions	Who: all members of the five change coalitions How: Four online meeting of 3 hours When: one meeting in between learning expedition one and two and two meetings after the learning expeditions in Q1, Q2 and Q3 2021 Tools & working methods: learning sessions/lessons sharing, feedback, carry forward of learning. What: The coalitions had separate meetings with members of the project team in between the learning expeditions and twice after the expeditions were finished, to discuss the implications of the expedition with each other, and to further elaborate their plans. In these meetings, each coalition received coaching on their role and task as a change agent by members of the project team. Themes that were covered and discussed during these coaching sessions were issues such as dealing with change uncertainty, handling resistance, and maintaining the focus on the desired behavioural change.

5. Training case conceptualisation	Who: Two members of each change coalition [n = 10] How: Four meeting of 3,5 hours When: Q3 2021 Tools & working methods: formal training [7-factors model] What: Ten participants received in-depth training about how to utilise the 7-factors model while performing a clinical case conceptualisation.


The implementation of integrated care took place during the Covid-19 pandemic. Therefore, it was impossible to organise live meetings. This was challenging, since our implementation strategy was mostly focused on a bottom-up approach with the aim of bringing professionals together to design the change to integrated care. We used multiple online tools to organize the online meetings. Similarly, the platform ‘Studytube’ informed professionals with literature and videos about integrated care to prepare the online meetings. Fortunately, with this approach, we were able to sufficiently engage and motivate professionals to join the project, despite the severe Covid-19 measurements.

No ethics approval was obtained for the study as we evaluated an internal quality improvement trajectory within our own organisation.

## Research methods

We used a mixed-methods approach because it yields a richer description of the phenomenon and the opportunity to corroborate and deepen our findings from different angles [27].

During the project, we consulted with our organisation’s client council ‘YouSay’ on several occasions to ask them to critically review the steps we were taking in the change toward integrated care. For example, when we were planning to implement the 7-factors model into our electronical health record system, we explicitly asked their opinion about this.

### Selection of study participants

We used a purposive sampling method – maximum variation sampling- to involve a wide range of perspectives and to gain greater insight by considering our research topic from various angles. This was also our criterion for accepting study participants. All the professionals from the five change coalitions [total n = 33] were invited to take part in the research. All agreed to participate in the research project verbally. These coalitions consisted of team leads, psychologists, care coordinators, youth care professionals, and an account manager with different roles in the change process [see Table 2]. The larger regions also have larger coalitions based on the number of FTEs per region. The five regions are comparable in the sense that they all provide integrated care in the same manner. Furthermore, in every region the same expertise is represented in the teams.

**Table 2:** Member composition of the five change coalitions.


FUNCTION	ROLE IN THE CHANGE PROCESS

**Change coalition 1**	

Teamlead [n = 2]	Enabler

Psychologist [n = 1]	Informal leader

Psychologist and care coordinator [n = 1]	Informal leader

Account manager [n = 1]	Hotspot leader

Youth care professional [n = 4]	Informal leaders

**Change coalition 2**	

Teamlead [n = 1]	Enabler

Psychologist [n = 1]	Hotspot leader

Youth care professionals [n = 5]	Informal leaders

**Change coalition 3**	

Teamlead [n = 1]	Enabler

Care coordinator [n = 1]	Hotspot leader

Youth care professionals [n = 2]	Informal leaders

**Change Coalition 4**	

Teamlead [n = 1]	Enabler

Psychologist [n = 1]	Informal leader

Care coordinator [n = 1]	Hotspot leader

Youth care professionals [n = 3]	Informal leaders

**Change Coalition 5**	

Teamlead [n = 2]	Enabler

Psychologist [n = 1]	Hotspot leader

Psychologist and care coordinator [n = 1]	Hotspot leader

Youth care professionals [n = 3]	Informal leaders


Note: Youth care professionals are professionals with a higher vocational level of education in social work or pedagogics, who provide care to the families in the regions.

### Operationalisation and assessment of behavioural change

One year after the program started [in October and November 2021], the behavioural changes of the participants were assessed in a structured group interview lasting approximately 90 minutes. The group interview protocol [see Appendix A, Q1–Q4] consisted of a set of questions to assess the frequencies of the four target ‘integrated care behaviours’, namely:

1. Reflecting on cases with colleagues with different expertise in a multi-disciplinary setting.
2. Performing a case conceptualisation to determine which care is most appropriate for a family.
3. Discussing this conceptualisation in the multi-disciplinary team to consider the family’s case from different areas of expertise.
4. Supporting colleagues in making the change towards working in teams with different expertise.

Scoring took place on five-point Likert-type scales to indicate the frequency of targeted key behaviours in the present and past.

The group interview thus applied a so-called retrospective post-then design [28,29] as follows. Firstly, participants were asked to discuss and estimate their success in carrying out targeted behaviours at present, after the change interventions were implemented. They were then asked to reflect on the extent to which they showed these behaviours prior to the intervention [i.e., a retrospective then-measure]. Next, the researchers facilitated group-dialogue to reach a reliable shared estimate of their current and previous behaviours, meaning their relative progress in behavioural change.

An independent reliability check on the group’s own assessment of their behavioural change was then carried out. The results of the group interview were discussed in a separate session with an independent observer of the group’s development. The independent observers were also employed at Youké and responsible for providing integrated care in the regions of each of the change coalitions. But they were not a part of the change coalitions, meaning they were not involved in shaping and carrying out the change to integrated care and can be seen as outsiders to the intervention. These independent observers were three psychologists and two youth care professionals. Specifically, they were asked to [dis]confirm the extent of change in behaviour that the change coalitions claimed to have achieved [See Appendix A, Q10]. All observers rated the accuracy of the groups’ own assessments of behavioural change from reliable to fairly reliable.

### Assessment of enabling and hindering factors

Following-up on the scaling questions, a group dialogue on five open questions [see Q5–Q9 in Appendix A] was facilitated. This served to deepen our understanding of which factors specifically helped or hindered the behavioural changes. We also asked how much confidence respondents had in actually realising the target behaviours.

## Data analysis

### Quantitative: scale questions on target behavioural switches

The degree to which each key behaviour was displayed in the five coalitions before and after the change intervention was measured. To assess statistical significance of behaviour differences before and after the change intervention across teams, the Wilcoxon Rank Sum Test was applied.

### Qualitative: open questions on hindering and enabling factors

Qualitative thematic analysis based on the steps described by Boeije, & Bleijenbergh [2019] and Maquire, & Delahunt [2017] was conducted on the open-ended questions [30,31]. Firstly, the researchers carried out in-depth readings of the group interview transcripts to gain familiarity with the data. Then, initial codes were generated by the researchers. To guarantee the quality of the analysis and the accuracy of the codes, all interviews were independently coded by the first author and second author. Discrepancies in coding were discussed until consensus was reached. Based on the initial codes, a codebook was developed. In a second coding round, axial coding took place to find overarching themes. All codes that were administered to an overarching theme were then reevaluated between coders. If the code did not fit the theme well enough, it was moved to another theme. The final themes were identified as the main key factors facilitating or hindering the change to integrated care. The data analysis was carried out in Dutch. Particularly relevant quotes were translated to English and included in this paper.

## Analysis and Results

### Quantitative results on behavioural changes

#### Target behaviour 1: Reflecting on clinical cases with colleagues with different expertise in a multi-disciplinary setting

**Figure 2:**
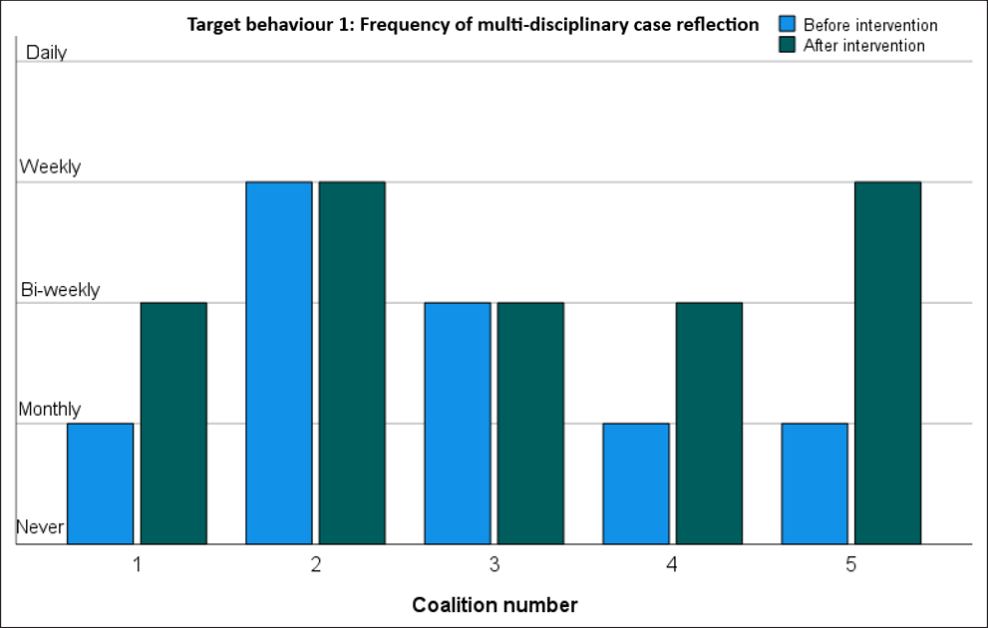
Frequency of multi-disciplinary case reflection.

Figure 2 shows the frequencies by which the five change coalitions reflected on cases in a multi-disciplinary setting before and after the change interventions. In three of the five coalitions the frequency went up as intended, even substantially so in coalition 5. No [further] change was recorded in coalitions 2 and 3, that already were at a relatively higher frequency of multi-disciplinary reflection.

#### Target behaviour 2: Performing a 7-factor case conceptualisation to determine which care is most appropriate for a family

**Figure 3:**
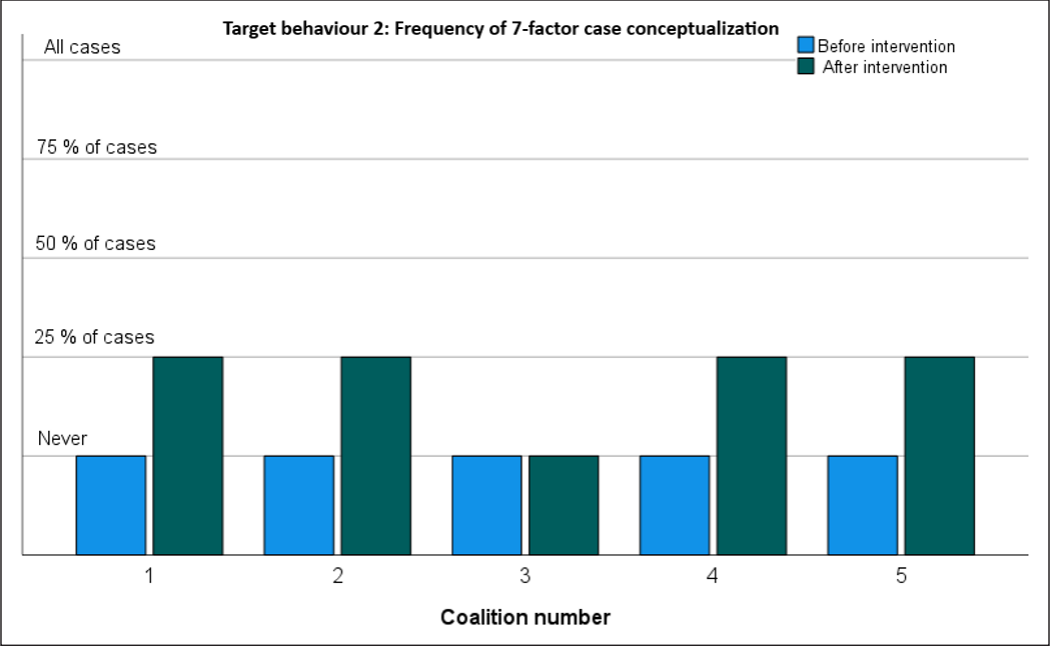
Frequency of 7-factor case conceptualization.

Figure 3 shows the extent to which professionals of the five change coalitions started to perform case conceptualisations based on the 7-factors model after the case method training intervention. Four of the five coalitions indeed started to implement this method on a limited scale [25% of the cases]. Only coalition 3 did not.

#### Target behaviour 3: Discussing the 7-factor case conceptualisation in the multi-disciplinary team to consider the family’s case from different areas of expertise

**Figure 4:**
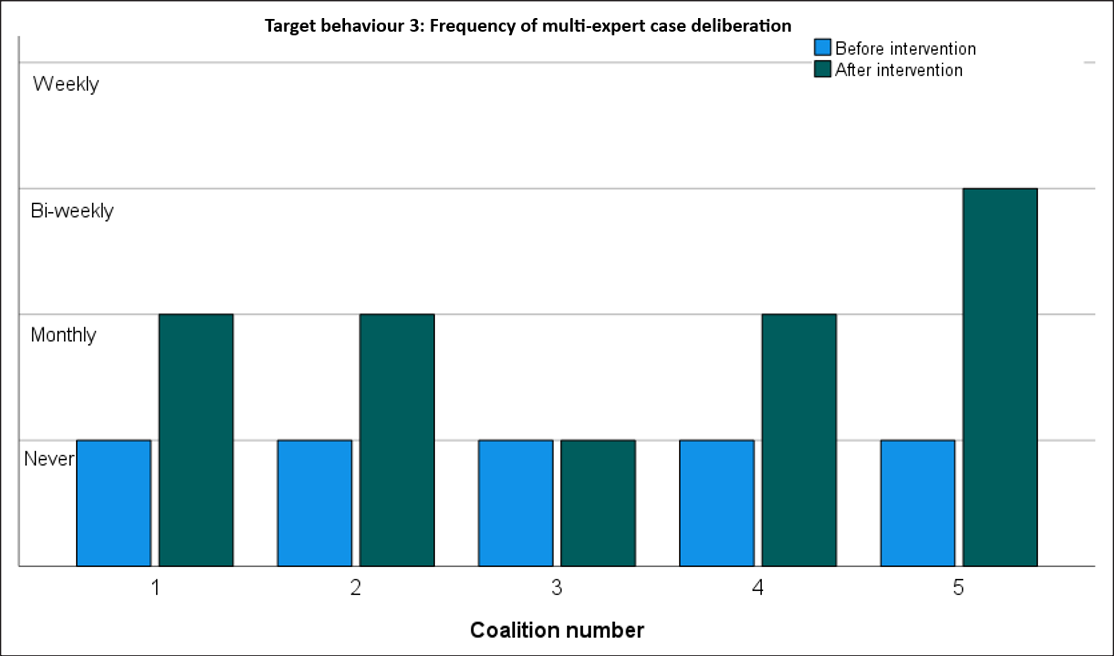
Frequency of multi-expert case deliberation.

Figure 4 shows the extent to which the five change coalitions actually discussed the 7-factor case conceptualisation in a multi-disciplinary setting to consider the family’s case from different areas of expertise. In line with the previous result [see Figure 3], all four coalitions that started using the method did indeed use it more frequent during multi-expert deliberations – coalition 5 even on a bi-weekly basis.

#### Target behaviour 4: Supporting colleagues in making the change towards working in teams with different expertise

**Figure 5:**
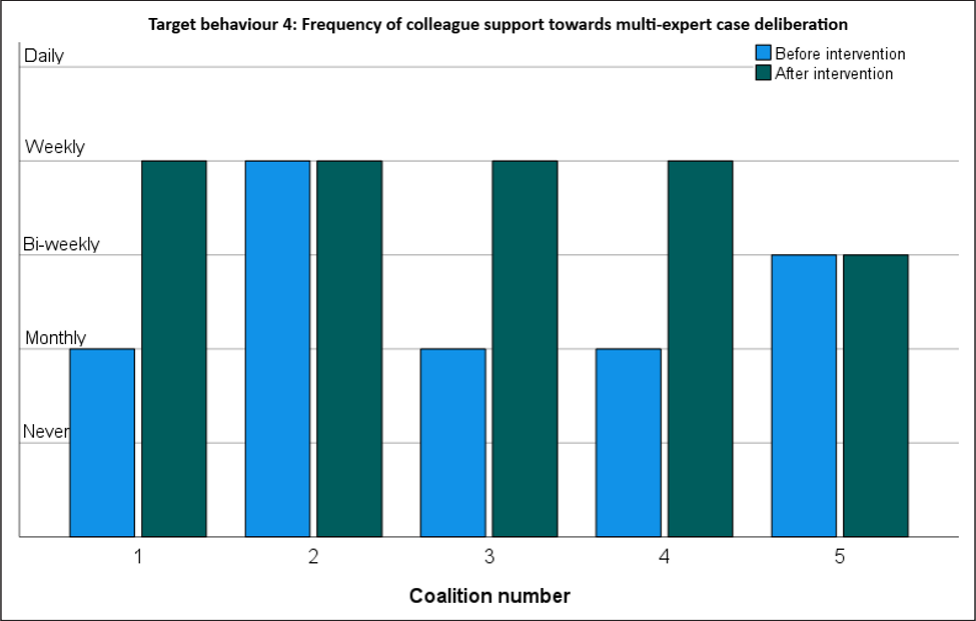
Frequency of colleague support towards multi-expert case deliberation.

Figure 5 shows the extent to which the five change coalitions increased support for their colleagues in realising the behavioural changes in daily practice. Three coalitions reported a substantial increase, moving from monthly to weekly colleague support. The other two coalitions started from already relatively higher levels of colleague support and reported no increase.

Overall, all coalitions reported increases in target behaviours. This was corroborated in a Wilcoxon Rank Sum test showing a significant increase [p < .000] in target behaviours across all change coalitions. As shown in Figures 2,3,4,5, the largest improvements occurred in coalitions 1, 4 and 5. Modest increases were recorded for coalitions 2 and 3. Coalition 2 started at an already relatively high level of target behaviour frequencies and may have had least to gain from the interventions. Coalition 3 seems to have improved only very modestly. It did not seem to pick up the new 7-factors model at all but did nevertheless report a substantial increase in supporting colleagues towards integrated care.

### Qualitative interview results on the overall change process

The open interview answers [Q5–Q9; see Appendix A] were content coded in order to get a deeper understanding of what helped and hindered daily progress in the change process. This yielded five broad themes. The first two relate to how the method supported the change and how individuals’ preconceptions might be a hindrance. Next, two important secondary process gains from the change-effort were identified. Finally, some effective preconditions for this approach were pointed out. These five themes are described in more detail below.

#### THEME 1 – Enablers stemming from the method: developing a clear path to change

A first set of factors that proved helpful in the change process had to do with characteristics of the project approach that clarified the path to change. We identified three key ingredients of this: forming a strong coalition to carry the change, establishing a sense of need and the importance of open dialogue to create commitment to behavioural change.

a. Forming a strong change coalition

The respondents indicated that the workforce needs a group of professionals such as the change coalitions who are responsible for ‘carrying’ the change. The members of the change coalitions should be intrinsically motivated for this task. They need a clear assignment and mandate from the organisation. Teamleads are considered crucial for the acceptance of the change and the group of ambassadors for facilitation and monitoring of the change process.

*“I can imagine it’s necessary for a number of people to be involved, possibly in different formations over time, to keep the change process active. Otherwise, everyone will just revert to their usual ways” CC 1, Psychologist]*

b. Establish a shared sense of perceived benefits

Most professionals felt a strong and urgent need towards the change path to integrated care. They thought it would benefit their own collaboration process with colleagues by giving them a deeper understanding of other expertise. Furthermore, they also strongly felt that families would receive quicker access to the most appropriate support.

*“Yes, families are helped in the most appropriate way, meaning fewer families on the wait list, and we can solve it together as one team. I hope that they [families] will receive intensive, combined care and are able to finish their treatment sooner than the current way.” [CC 1, Teamlead]*

For a change project to succeed, professionals thought it necessary that everyone involved saw the need to, and benefits of the change. Professionals seemed to be more inclined to change their behaviour if they felt the need to do so. However, some professionals did not feel this need to change. In case of doubts, the importance of the change was repeated and discussed in coaching sessions or in informal bilaterals with the project lead and the policy advisor.

c. Organising open dialogue to build behavioural change commitment

The change coalitions organised two meetings with their colleagues to shape the change within their region. All professionals working in a region were invited for these meetings. The meetings provided plenty of opportunities to share their opinions about how they wanted to shape the change. This was seen as beneficial to the change process as it went beyond just giving information about the process from a top-down perspective. Discussing doubts and questions about the change process with their colleagues in the field is considered very important.

“*There was a lot of space for giving and discussing feedback. It wasn’t a meeting to just convince people of something or to nudge them into a certain direction. It was clear that something was about to change but there was a lot of room to discuss how things would change*.” [CC2, youth care professional]

Clear agreements on how the new behaviour should be expressed guaranteed everyone had the same concept of it. Experimenting with new ideas in small groups to discover effective strategies in the change to integrated care, and celebrating successes to build commitment were considered as helpful in the change process.

#### THEME 2 – Hindrances: obstructive beliefs and behaviours

Fear and resistance to change often had to do with unhelpful preconceptions and old habits. Three subthemes were identified in this category.

a. Fear of losing one’s own expertise

Changing from working exclusively with colleagues from the same expertise to working in an integrated team with professionals from different fields of expertise, led to a fear of losing expertise in some professionals. They feared that by letting go of their old way of working, they would lose [part of] their professional identity. Furthermore, they were afraid of professional distraction, of not being able to keep up with developments in their own field of expertise. As a result, some professionals mentioned they would still rather consult with colleagues from their own field of expertise, because they believed it would benefit them more.

b. Reluctance to let go of old habits

Although most of the professionals felt a sense of need for the change towards integrated care, professionals who were very satisfied with the current situation did not feel the need to change. This led to a lack of motivation for making the change to integrated care. Specifically, these professionals reported finding it difficult to let go of old habits, because the consequences of the change were not appealing to them and were not perceived as urgent or even necessary.

“*I think that a lot of professionals were really satisfied with the way things used to be organised. […] They are not particularly pleased with the change*.” [CC 5, Teamlead 1]

c. Negative experiences with prior change

Believing that change ultimately and inevitably leads to loss makes it difficult to change behaviour and habits or commit to the change process. This belief was strengthened for some professionals who actually experienced similar changes in other organisations. In these past experiences, some professionals ended up in an unsatisfactory work environment and even terminated their employment.

“*All those factors ultimately consist of one thing: The belief that change will result in something negative. That belief makes change complicated*.” [CC 5, Teamlead 2]

#### THEME 3 – Secondary process benefits: increased experience and appreciation of collaboration benefits

Most professionals experienced added value in collaborating with colleagues from different disciplines. They felt the opinions and advice of these colleagues were overall beneficial to determine the most appropriate care for families. According to professionals, being highly qualified and specialised in a certain expertise is important but has the risk of exclusively considering cases from this specific, and potentially limiting view. Considering a case from multiple viewpoints was seen as contributing to the collaboration process. Additionally, professionals felt valued and supported by colleagues with different expertise, and they experienced increased job satisfaction. Also, professionals felt these positive experiences caused them to focus on fostering and sustaining this new way of collaboration.

However, some professionals were hesitant and doubtful about the change, and still wondered whether the new way of working was to be preferred to the old one. At least, a need for further experience-building seems warranted.

#### THEME 4 – Secondary process benefits: increased and easier knowledge integration

Most professionals were very enthusiastic about gaining knowledge and insight into the expertise of their colleagues. According to them, this was caused by the change to integrated care and the new way of collaboration. Professionals felt it was far easier than before to ask other professionals for advice and guidance, simply because they were more familiar with each other’s expertise and what they could potentially gain from it. Furthermore, professionals indicated that the increased familiarity diminished certain thresholds to asking colleagues from a different field of expertise for advice.

#### THEME 5 – Boundary conditions for change

Professionals shared their ideas about which preconditions they deemed crucial in realising the change to integrated care in daily practice. These had to do with collectively building a new routine, having a good grasp on the time available to make changes, and receiving appropriate training.

a. Build routine in use of the 7-factors model

Performing a case conceptualisation using the 7-factors model and discussing this in a multi-disciplinary team was deemed quite complicated, because it demands implementing a whole new routine. Professionals mentioned that building routine in using a model for this conceptualisation, especially in complex cases, is necessary to realize a smooth collaboration in team meetings.

“*And that routine isn’t there yet. It’s a change in behaviour, which has to come from within*.” [CC 4, Teamlead]

b. Plan and allow sufficient time

Both the process of change itself and making the four behavioural switches were described as very time intensive. An important precondition is to know the amount of time professionals are allowed to invest, as some professionals felt pressure to meet production demands while simultaneously working on the change.

“*When you start working with a model like this, in the beginning it will take a lot of time because it’s something new, maybe you’re not completely convinced yet that this is the best suited model, and because you’re used to other ways of working*.”[CC 1, youth care professional]

c. The importance of training

According to several teamleads, training in performing clear case conceptualisations is important to work from a common ground. Professionals described that training is best given at the team level, so that it will also promote interprofessional collaboration within a team.

“*I really feel training is important, so everyone acts from the same understanding. If someone is struggling in the team training allows us to help each other out*.” [CC 4, youth care professional]

## Discussion

This study focused on a change process towards an integrated approach in Youké, an organization for specialised youth service. The careful formation of the change coalitions [22] was deemed very helpful in starting the change process within the organisation. Kicking off by discussing the ‘why’ of the change [26] ensured all the change coalitions felt motivated to start the change process and the need of the change was made clear. Members of the change coalitions can be seen as ‘early adopters’ [32] who subsequently, through coaching of the project team, engaged other professionals within the organisation to follow in their footsteps. This dialogic approach uses the energy and drive of the organisation’s ‘grass roots’ – the professionals – instead of forcing the change from the top down [23]. It also increased professionals’ awareness of the need for change. Through dialogue, we aimed to define the desired change in collaboration with professionals from all layers of the organisation. The change was made ‘real’ and practical by repeatedly asking the workforce to envision the change in terms of specific new behaviours to be learned. As such it immediately made direct sense for their daily practice. This focus on behavioural change also helped professionals to become more reflective on the way they functioned as a team.

Such increased ‘team reflexivity’ is known to be linked to effective team performance [33]. Research shows that transition periods -as in our case study- are optimal moments for reflection on achievements, providing ample learning opportunities to determine where improvements can be made [34,35]. The intervention described here may therefore indirectly foster more effective learning for teams in a more general sense. It builds a team competency even apart from the intended change. This may explain the experiences of coalition 3, where the frequency of the targeted behaviour hardly changed, but where the level of colleague support increased substantially due to the increased collective awareness and reflection on their behaviours. Such inadvertent ‘by-catch’ is very valuable.

Although professionals subscribed to the need for more integrated care delivery and experienced added value in collaborating with colleagues from different fields of expertise, some experienced a lot of uncertainty. They professed to be missing clear guidelines in policy about how to interpret the change. Apart from such cognitive difficulties -possibly explainable by a lack of proper information [36] – a second, more emotional struggle manifested. This was predominantly caused by ingrained professional beliefs and behaviours [36]. In line with previous research [12], we found that professionals were afraid to lose their expertise and/or felt their professional identity was being threatened. This provides a very clear area of attention in forming integrated care teams. Attention should be given to forming an additional team sense of identity, emanating from being a responsible cross-functional professional. Again: this should be accomplished by lots of dialogue but also through proper example-setting and role-modelling by prestigious colleagues from within the expert’s community. Only they can lend credibility and legitimacy to these new role-aspects.

Clear professional roles and realistic expectations are important facilitators related to the professional identity when providing integrated care. Moreover, we found that each shift of behaviour asks for numerous small adjustments in daily practice that can lead to feeling overwhelmed. It is helpful to have a shared learning mindset in order to ‘hold’ these inherent uncertainties. Finally, there was some resistance reported stemming from a lack of trust due to previous organisational experiences. It was therefore deemed important that professionals could openly voice their concerns based on previous experiences and draw proper lessons from those experiences in order to improve.

Directed collective behavioural change is a social learning process and our change project clearly reflects Kolb’s classic cycle-model of experiential learning [37]. Kolb describes how collective learning occurs through a combination of thorough and collective reflection and behavioural experimentation. The cycle consists of 4 stages, which in our change project looked as follows:

1. *Active experimentation* when we first started to implement new working methods, e.g. changing teams to collaborative, multi-disciplinary teams of professionals with various expertise and multi-expert case deliberation while using the 7-factors model as first ‘prototypes’ in daily practice.
2. Gathering *concrete experience* with the new behaviours, e.g. leading and coaching colleagues toward the change to integrated care.
3. *Reflective observation*. While experimenting we experienced and discovered which factors were facilitating [e.g. gaining familiarity with each other’s expertise] and which factors were hindering change [e.g. fear of losing own expertise and professional identity]. We are currently in the stage of reflecting on what we gained from this change and what we have yet to accomplish – this paper is a result of this and the next step.
4. *Abstract conceptualisation*. In going through steps 1 to 3 we ‘learn about our learning’ and use the lessons drawn to adjust the implementation process and the way integrated care can be best organised in our local setting. This provides the basis for future experiments.

To foster the durability of the changes a number of ‘anchoring actions’ were taken after this study was completed. First, frequent communication on the changes that were made and the philosophy behind it, is now a core part of daily practice within Youké. Second, to support teamleads in leading the new integrated care teams, quarterly meetings are organised. Third, a group of ‘key users’ of the 7-factors model is organised every two months to support the workforce in adapting and using the model. Finally, these initiatives are facilitated and led by the policy advisor who is now program leader for integrated care and the project lead who is now a member of the management team. This will be helpful in making sure that providing integrated care is, and remains an important topic to be discussed and monitored in the future.

### Strengths and limitations

This change and research project took place during the Covid-19 pandemic which led to obvious challenges. Fortunately, the pandemic has not delayed implementation of the interventions nor the progress of the change project as a whole. To the contrary even: the crisis brought unexpected learning benefits. The restrictions on live meetings required adaptation of our research method to online formats. We developed well-structured online meetings and learned to use new online tools and platforms to facilitate professionals in engaging in the project. As professionals could participate in the meetings from home and travelling was not necessary, it lowered the effort of participation, and made it easier to include professionals from different regions.

Two limitations of this study are important to consider. First, voluntary professionals in a change coalition tend to be skewed toward being supportive early adopters [32]. Consequently, there is a reasonable chance that these professionals have a more-than-average positive outlook on the change efforts and its effects. However, introducing change in a planned way typically starts -and should arguably start- with ‘the willing’, creating an inevitable mismatch between desired set-ups for effective change and those for optimal research validity.

A second limitation is the modest scale of the study. The effort of close monitoring of actual behavioural change within teams practically restricted the number of teams to be included in this project. Finally, a post-then design to measure intervention effects is arguably not among the strongest design choices. However, we strove to secure the reliability and validity of the results in our project by focusing on actual, observable behaviour towards integrated care, instead of merely the opinions and beliefs of participants. By using scale questions, the participants were given anchors to judge the extent in which their behaviour had changed.

This means that the results of our case study need to be interpreted with caution and can only be generalised to comparable organisations and contexts. Future research may focus on whether integrated care does indeed increase the quality of care and decrease fragmentation in care based on the perspective of families. Additionally, it could be interesting to explore how to actively involve families in shaping and carrying out change projects within organisations like Youké, enabling them to contribute to the expansion of knowledge based on their own lived experience.

## Conclusion

Our case study yields practical insights for concrete change interventions that can be helpful in implementing integrated care within a youth care organisation. We found factors contributing to behavioural change of professionals in providing integrated care. During this process it was crucial for the success of the change interventions to continuously motivate and facilitate professionals to focus on the change process and making them aware what this meant for their attitude and behaviour in daily practice.

## Additional File

The additional file for this article can be found as follows:

10.5334/ijic.8842.s1 Supplementary files. Appendix A and B.
