# Supplementary material for: Fostering Behavioural Change Towards Integrated Care – a Multi-Team Case Study in Specialised Youth Services
Source: Int J Integr Care. 2025 Feb 7;25(1):7. doi: 10.5334/ijic.8842 (PMC11804174; doi:10.5334/ijic.8842)
Supplement: Supplementary files. — Appendix A and B. [file ijic-25-1-8842-s1.pdf]

Supplementary files

Appendix A. Questionnaire protocol

The project integrated youth care was aimed at realising collaboration with colleagues with different expertise in a multi-disciplinary setting, performing a case conceptualisation to determine which care is most appropriate for a family, and discussing this conceptualisation in the multi-disciplinary team to consider the family's case from different areas of expertise.

Please read the following statements. Each statement is shown twice. The first time we want you to state your current behaviour. The second time we want you to state your behaviour before the change coalition started. Below the statements you will find five categories to choose from. [This questionnaire protocol was administered in Dutch and is translated to English for this paper.]

Scale questions

Consider your current behaviour:

Q1 I discuss clinical cases with colleagues with different expertise in a multi-disciplinary team setting.

Scale Never – Once a month – Once every two weeks – Once a week – Daily

Recall November 2020, before you joined the change coalition:

Q1 I discussed clinical cases with colleagues with different expertise in a multi-disciplinary team setting

Scale Never – Once a month – Once every two weeks – Once a week – Daily

Consider your current behaviour:

Q2 I make a clinical case conceptualisation according to the 7-factors model

Scale Never – 25% of the cases – 50% of the cases – 75% of the cases - Every case

Recall November 2020, before you joined the change coalition:

Q2 I made a clinical case conceptualisation according to the 7-factors model

Scale Never – 25% of the cases – 50% of the cases – 75% of the cases - Every case

Consider your current behaviour:

Q3 I discuss a clinical case conceptualisation with colleagues with different expertise.

Scale Never – Once a month – Once every two weeks – Once a week – Daily

Recall November 2020, before you joined the change coalition:

Q3 I discussed a clinical case conceptualisation with colleagues with different expertise.

Scale Never – Once a month – Once every two weeks – Once a week – Daily

Consider your current behaviour:

Q4 I support/help colleagues to make the change towards working in teams with different expertise.

Scale Never – Once a month – Once every two weeks – Once a week – Daily

Recall November 2020, before you joined the change coalition

Q4 I supported/helped colleagues to make the change towards working in teams with different expertise.

Scale Never – Once a month – Once every two weeks – Once a week – Daily

Open ended questions

Q5 Which of the behavioural change shifts is the easiest? Why?

Q6 Which of the behavioural change shifts is the most difficult? Why?

Q7 What are the hindering and facilitating factors in discussing/reflecting about cases in a team that consists of professionals with different expertise? Why?

Q8 What are the hindering and facilitating factors in performing a clinical case conceptualisation and discussing this in the multi-disciplinary team according to the 7-factors model? Why?

Q8 What is needed to stick to the behavioural change shifts?

Q9 What should be seen when the change operation has an ultimate success? How far has it progressed?

Protocol interview independent observers

Q10 What is your estimation of the consensus reliability of the results for Q1, Q2, Q3 and Q4?

Reliable – Fairly reliable – Not reliable/Not unreliable - Fairly unreliable – Unreliable

Appendix B. Frequency of Main themes and subthemes

Table 3 Frequency of main themes and subthemes

Main themes	Subthemes	Frequency
**1.Developing a change path**		62
	A strong change coalition	14
	Sense of need for integrated care	9
	Building commitment for behavioural changes	39
**2.Ingrained beliefs and behaviours**		21
	Fear of losing expertise	13
	Letting go of old habits	5
	Negative experiences with change	3
**3.Adaptation of change in daily practice**	-	28
**4.Knowledge of each other’s expertise**	-	40
**5.Preconditions for change**		48
	Routine in use of model	19
	Time intensity	8
	Importance of training	10
	Importance of structure in team meetings	11
